# Does the loot box open the door to addiction? A case report of gaming disorder with high charges for loot box purchases

**DOI:** 10.1002/pcn5.167

**Published:** 2024-01-18

**Authors:** Tokuya Inaguma, Sumiko Misumi, Tomoyuki Funatogawa, Takahiro Nemoto, Hirohiko Harima, Masafumi Mizuno

**Affiliations:** ^1^ Department of Psychiatry Tokyo Metropolitan Matsuzawa Hospital Tokyo Japan; ^2^ Department of Neuropsychiatry Toho University Graduate School of Medicine Tokyo Japan

**Keywords:** addictive behavior, gaming disorder, gambling disorder, loot box, reward system

## Abstract

**Background:**

A loot box is a gaming term for an electronic lottery that randomly provides items that enhance the gaming experience. In recent years, loot boxes have been increasingly discussed as a risk factor of gaming disorder (GD). While they may be purchased for a few dollars at a time, the cumulative expenses resulting from their addictive use have become a social problem.

**Case Presentation:**

This paper presents a case of GD involving a substantial financial burden incurred through the use of a Japanese loot box called Gacha.

**Conclusion:**

The randomness in the selection of virtual items in loot boxes resembles gambling, triggering the reward system and contributing to an addiction to purchasing more loot boxes. For therapeutic purposes, understanding the motivations behind purchasing loot boxes and considering individual developmental characteristics are crucial to helping patients find satisfaction and a sense of achievement in activities besides gaming.

## BACKGROUND

Gaming disorder (GD), which is related to gambling disorder, was officially included in the ICD‐11 as a group of disorders due to addictive behaviors.^1^ Recently, the loot box, a virtual item purchased to enhance the gaming experience, has emerged as a discussed risk factor of GD.^2^ These loot boxes randomly offer items for purchase to players,^3^ attributing a perceived scarcity to certain virtual objects labeled as “rare.” While these loot boxes may be purchased for a minimal amount, such as a few dollars each time, the cumulative expenses incurred through their addictive use have become a societal concern.^4^ Moreover, the random nature of loot boxes can lead to gambling.^5^ This report details the case of a GD patient hospitalized after extravagant spending on loot boxes.

## CASE PRESENTATION

An unemployed man in his early 20s presented with the chief complaint of being unable to stop playing video games. He was the second child of three siblings and lived with his parents and younger brother. He had no history of perinatal abnormality but made his first utterance at 18 months. While at nursery school, he was restless and unable to pay attention to the nursery school staff. He was very interested in insects. As a public elementary school pupil, he was often forgetful. Owing to his deficient communication skills and high impulsivity, he became isolated and gradually stopped attending school. At the age of 11 years, he received the diagnosis of attention deficit hyperactivity disorder (ADHD) and autism spectrum disorder (ASD) at a local clinic. Since then, he has been receiving support for his developmental challenges. Atomoxetine was initiated but discontinued due to side effects. After graduating from a public junior high school, he entered a private high school. In his third year, under the pressure of studying for university entrance examinations, he began playing video games on his smartphone.

In Year X−1, he enrolled in a university but struggled to keep up with his courses and failed to get along well with his peers. These experiences led to feelings of inferiority, which he attempted to relieve by playing video games. He found satisfaction in obtaining rare loot box items. However, he was unable to control his gaming behavior and played at all hours of the day and night, eventually dropping out of university. He gained weight (body mass index [BMI] 32.2) from lack of exercise. Unable to control his craving to purchase items from the loot box, he secretly used his mother's credit card and threatened her with violence when he was discovered. He spent several thousand dollars a month on these purchases. In Year X, he presented to our hospital with the chief complaint of being unable to stop playing video games.

The autism‐spectrum quotient (AQ)^6^ and the Wender Utah Rating Scale (WURS)^7^ were administered. The patient obtained an AQ score of 35 and a WURS score of 68, both of which were above the cutoff.

His tripartite diagnosis, based on the ICD‐11, consisted first, of GD, predominantly online (6C51.0), characterized by impaired control over gaming behavior, increased priority given to this behavior, and its persistence despite negative consequences, such as academic failure, high financial costs, violence, and weight gain; second, ADHD with predominantly hyperactive‐impulsive presentation (6A05.1) as seen in the persistent pattern of inattention, hyperactivity, and impulsivity; and third, ASD (6A02), which was diagnosed on the basis of his persistent inability to sustain social communication and restricted, repetitive, and inflexible behavior patterns.

The patient agreed upon a 1‐month hospitalization period. Digital detox therapy, aimed at reducing time spent playing video games, was initiated. After discussion, the patient consented to limit gaming to just 1 h per day. Although anti‐ADHD medications were considered for his impulsivity, he declined the treatment owing to his experience of the adverse effects of atomoxetine.

Subsequently, the patient received psychoeducation about the basics of GD. For example, he learned that GD is an addictive behavior arising from control impairment, it becomes more challenging when coupled with ADHD, and it negatively impacts daily life yet serves as a compensatory mechanism against daily stress. Moreover, the necessity to seek satisfaction and a sense of accomplishment through activities other than gaming was highlighted. While acknowledging the negative impact of his gaming behavior, the patient admitted struggling to control it.

After 1 week of hospitalization, he became irritable and complained about the restrictions placed on his gaming habit, saying, “I really did not want to be hospitalized” and “I get very frustrated.” This irritation was considered a potential symptom of withdrawal. The patient began consuming excessive amounts of snack food to distract himself. When advised to reduce snacking to prevent weight gain, he threatened to break the windows in his ward unless he was continuously supplied with snacks. This response mirrored the previous threatening behavior toward his mother when she objected to his loot box purchases. In response, the patient was informed that threats would not be tolerated and was offered a reward system, receiving snacks for 30 min of stationary bicycle exercise. Despite initial objections, he complied and seemed to relish the achievement of losing weight. Additionally, relaxation techniques like deep breathing and muscle relaxation were taught to manage his irritation.

To reinforce treatment adherence, a token economy was instituted whereby the patient received stickers for following restrictions. He was rewarded with a walk with a nurse after accumulating 10 stickers. Although he sometimes requested additional time to play video games, he generally adhered to the regimen. Collecting stickers seemed rewarding, akin to acquiring items from a loot box. Occupational therapy sessions were encouraged to broaden his range of activities besides gaming. Despite sometimes asking to be excused from such activities, he participated daily. Staff consistently praised his progress, which seemed to satisfy him greatly.

The patient's parents received educational sessions about GD pathology and their child's developmental characteristics. They learned how to manage their child's spending habits by setting a limit on his allowance money with his consent, not to give into demands for more money even if threatened, and to communicate clearly that violence was unacceptable. They were advised to motivate the patient to participate in activities besides gaming and to praise him for even the smallest progress.

At the end of the hospitalization, he was praised for his persistence in managing his frustration as well as for his efforts to engage in activities besides gaming. He recognized that he could shorten his gaming time by scheduling activities. Specifically, he acknowledged that his exercise routines could contribute to weight loss (BMI 32.2 → 31.6), alleviate frustration, and foster a sense of achievement.

After 1 month's hospitalization, he was discharged. Thereafter, he began exercising at a gym and found satisfaction in getting into shape. He also began working parttime, saying, “I want to increase the amount of money I can spend freely.” On workdays, he left his smartphone at home. His gaming time was reduced to about 3 h a day. His charges dropped to $500 a month, which he was able to pay using the income from his parttime work. He no longer threatened his mother. A year after discharge, he continues to work parttime and seems to have succeeded in reducing the priority of gaming, saying, “I think it's time for me to quit playing video games.”

## DISCUSSION

GD, which is related to gambling disorder, was officially included in the ICD‐11 as a group of disorders due to addictive behaviors.^1^ The cardinal symptoms of GD are impaired control over gaming behavior, increased priority given to the behavior, and its persistence or escalation despite negative consequences to the patient.^8^

In the present case, one major reason for the patient's loss of control over his gaming behavior was likely to be the element of gambling inherent in the loot box. There is a strong resemblance between getting rare items in loot boxes and taking a gamble.^9^ As players randomly obtain items from loot boxes, they become compelled to purchase more loot boxes in the expectation that they will eventually obtain a desired item. This is known as the variable ratio reinforcement schedule.^10^ This powerful behavioral reinforcement mechanism underlies the addiction to gambling.^11^

Uncontrolled gaming and gambling behaviors parallel the mechanisms of substance dependence.^12^ Extensive analysis comparing current video games with gambling may reveal common biological mechanisms underlying behavioral addictions and substance dependence.

The young man in the present case spent a large amount of money repeatedly purchasing loot boxes. Indeed, this type of extravagance associated with gaming has become a social problem.^4^ Loot boxes are commonly purchased by many young people. The younger they are, the more difficult it is for them to control their impulsivity, which makes them highly susceptible to gambling behavior.^13^ In other words, the loot box can become a gateway to gambling disorder in young people.^5^ Thus, appropriate regulation of this aspect of the gaming industry becomes crucial.

Regulations regarding loot boxes vary in each country. In Japan, legal restrictions on loot boxes have been published by the Consumer Affairs Agency.^14^ Additionally, the Japan Online Gaming Association has established guidelines on loot box use.^15^ However, the guidelines do not prescribe an upper limit on payments and fail to disclose the acquisition rate, therefore their effectiveness is limited in preventing large payments. China and South Korea mandate disclosing the probability of obtaining prizes.^16^ Belgium and the Netherlands consider loot boxes as a form of illegal gambling^17^ while the United States lacks uniform legislation on this matter.^18^

The present case involved the comorbidity of ADHD and ASD. The former, in particular, is associated with GD. Stimulus‐rich games aggravate ADHD traits, such as avoidance of delayed rewards, preference for immediate rewards,^19^ and proneness to boredom.^20^ Biologically, both GD and ADHD involve the reward system circuit^21^; dysfunction of the executive regulation network in ADHD may be a predisposing factor of GD. For this reason, gambling disorder is also associated with ADHD.^22^ In other words, games with a gambling element are even more difficult to control for patients with ADHD.

The token economy and positive feedback were effective in rehabilitating the present patient. These techniques correspond to the interventions recommended for ADHD.^23^ Previous studies have indicated a consistent association between symptoms of ADHD and GD.^24^ Compared to direct intervention for the behavioral addiction itself, interventions for comorbid ADHD may be more useful and/or necessary.^25^

Inpatient treatment was chosen in the present case. Alongside inpatient programs, Japanese psychiatric hospitals provide specialized daycare programs and therapeutic residential camps for treating GD.^25^ While some studies have shown their effectiveness,^26^, ^27^ further research is necessary.

## CONCLUSION

The present study discussed a case of GD involving a large financial burden resulting from using a Japanese loot box called Gacha. The chance‐based nature of loot boxes is similar to gambling. Obtaining the desired items randomly from loot boxes reinforces the reward system, leading to an addiction to purchasing them. Therapy should address the patient's motivation for purchasing loot boxes while considering individual developmental characteristics and guide the patient toward finding satisfaction and a sense of achievement in activities besides gaming.

## AUTHOR CONTRIBUTION

Tokuya Inaguma and Sumiko Misumi acquired case data. Tokuya Inaguma drafted the manuscript. All the authors revised the manuscript and have read and approved the final version.

## CONFLICT OF INTEREST STATEMENT

Takahiro Nemoto is an Editorial Board member of *Psychiatry and Clinical Neurosciences Reports* and a co‐author of this article. To minimize bias, he was excluded from all editorial decision‐making related to the acceptance of this article for publication.

## ETHICS APPROVAL STATEMENT

This case report was conducted in accordance with ethical guidelines for case reports of the Japanese Society of Psychiatry and Neurology.

## PATIENT CONSENT STATEMENT

Written informed consent was obtained from the patient for publication of this report.

## CLINICAL TRIAL REGISTRATION

N/A

## Data Availability

The data in this case report are available from the corresponding author (T.I.) upon request.
